# Internet survey on the actual situation of constipation in the Japanese population under 70 years old: focus on functional constipation and constipation-predominant irritable bowel syndrome

**DOI:** 10.1007/s00535-019-01611-8

**Published:** 2019-08-19

**Authors:** Yurika Kawamura, Sayuri Yamamoto, Yasushi Funaki, Wataru Ohashi, Kazuhiro Yamamoto, Tomonori Ozeki, Yoshiharu Yamaguchi, Yasuhiro Tamura, Shinya Izawa, Yasutaka Hijikata, Masahide Ebi, Naotaka Ogasawara, Makoto Sasaki, Kunio Kasugai

**Affiliations:** 1 Division of Gastroenterology, Aichi Medical University School of Medicine, 1-1 Yazakokarimata, Nagakute, 480-1195 Aichi Japan; 2 Division of Biostatistics, Clinical Research Center, Aichi Medical University School of Medicine, Aichi, Japan

**Keywords:** Constipation, Functional constipation, Irritable bowel syndrome, Rome III criteria, Internet survey

## Abstract

**Background:**

In Japan, the prevalence of constipation-predominant irritable bowel syndrome (IBS-C) and functional constipation (FC) diagnosed by the Rome III criteria is unclear, as are the demographic profile, quality of life (QOL), and habits of persons with IBS-C or FC.

**Methods:**

We performed an internet survey of constipation. After extracting 3000 persons fitting the composition of the general Japanese population, we investigated demographic factors, lifestyle, defecation, and laxatives. IBS-C and FC were diagnosed by Rome III criteria. Respondents also completed the Japanese IBS severity index (IBS-SI-J), Japanese IBS QOL scale (IBS-QOL-J), SF-8, Hospital Anxiety and Depression Scale (HADS), and Japanese Health Practice Index (JHPI).

**Results:**

There were 262 respondents with FC (8.73%) [73 men and 189 women; mean age: 49.8 ± 13.1 years; mean body mass index (BMI): 21.0 ± 3.3 g/m^2^] and 149 respondents with IBS-C (4.97%) (76 men and 73 women; mean age; 41.6 ± 13.7 years; mean BMI: 20.8 ± 3.0 kg/m^2^). Total IBS-QOL-J score were significantly lower in the IBS-C group than the FC group. With regard to SF-8, score of mental component summary (MCS) was significantly lower in the IBS-C group. The total IBS-SI-J score and item scores, except for satisfactory defecation, were significantly higher in the IBS-C group than the FC group. HADS showed a significant increase of anxiety and depression in both the groups, and the JHPI revealed insufficient sleep.

**Conclusions:**

In Japan, among the population of under 70 years old, the prevalence of IBS-C and FC (Rome III criteria) was 4.97% and 8.76%, respectively. IBS-C caused more severe symptoms than FC, resulting in impairment of QOL.

**Electronic supplementary material:**

The online version of this article (10.1007/s00535-019-01611-8) contains supplementary material, which is available to authorized users.

## Introduction

According to the 2016 Comprehensive Survey of Living Conditions conducted by the Japanese Ministry of Health, Labour and Welfare, the prevalence of constipation ranges from 2 to 5% in Japan and it shows a higher frequency in females than males (4.6% vs. 2.5%) [1]. However, the actual situation of constipation in the general Japanese population is unclear, including the methods used to control it, particularly if we not only consider patients receiving medical treatment, but also persons who control their symptoms with over-the-counter drugs and supplements.

Since constipation-predominant irritable bowel syndrome (IBS-C) and functional constipation (FC) were defined by the Rome criteria [2], many epidemiological studies have been conducted in Europe and the USA [3–6], but there have been few investigations of the actual status and profile of Japanese persons with FC or IBS-C [7–9]. In addition, effective countermeasures for constipation such as modification of the diet and lifestyle have not been established, and the selection criteria for medications to treat this condition remain unclear. Moreover, there have not been any studies comparing FC to IBS-C with regard to these points.

Therefore, investigation of the use of laxatives and characteristic countermeasures for constipation, the influence of constipation on the quality of life (QOL), and the lifestyle and exercise habits of persons with constipation could provide useful data for the development and assessment of therapeutic strategies. Accordingly, we performed an internet questionnaire survey of constipation in Japan and extracted respondents who had FC or IBS-C according to the Rome III criteria [2, 9, 10], with the objective of determining the characteristics of these two groups with different types of constipation in the Japanese population.

## Methods

From October 8 to 11 in 2016, a preliminary internet questionnaire was completed by 10,000 Japanese panelists with constipation aged 20–69 years. Hospital admission and use of medications were not considered as inclusion/exclusion criteria. Men or women who gave informed consent to participation were enrolled in the internet survey. The following persons were excluded from the survey:

1. Persons with a history of abdominal surgery, other than appendectomy.
2. Persons with small and large intestinal diseases, such as inflammatory bowel disease (ulcerative colitis or Crohn’s disease).
3. Persons with bowel cancer or other cancers.
4. Women who were pregnant.
5. Persons with a history of gastric/intestinal disease, such as gastric or duodenal ulcer, hemorrhoids, diverticulitis, or diverticulum.
6. To exclude secondary constipation, persons with a history of cerebral infarction were ineligible, as were persons with neurological disease, chronic obstructive lung disease, hepatic disease, or renal disease.
7. To exclude drug-induced constipation, persons with diabetes using oral antidiabetic agents or insulin were ineligible, as were persons with hypertension using antihypertensive agents, and persons taking chalybeate (mineral spring water), hypnotics, sedatives, or antipsychotic agents.
8. Persons who were unable to correctly follow the instructions for completing the survey.

Panelists who refused to answer the questionnaire or failed to complete it were classified as dropouts.

**Fig. 1:**
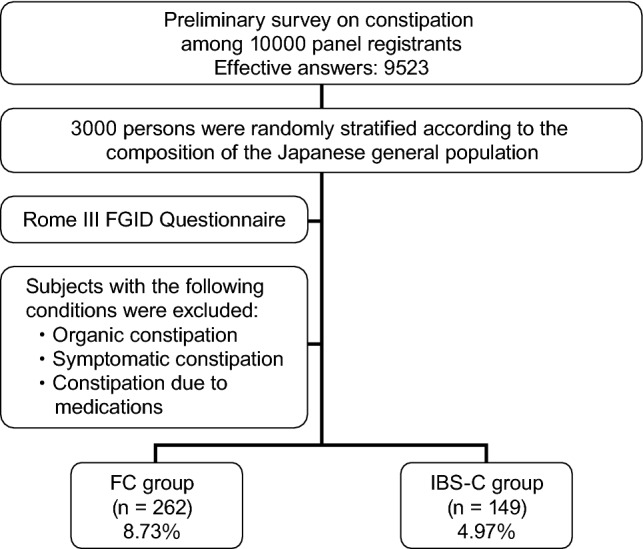
Study outline

Questionnaires with sufficient data for analysis were obtained from 9523 persons. Among the 4909 persons who responded to the question ‘Do you think you have constipation?’ (on a 5-item Likert scale) by selecting ‘I strongly think I have constipation’ or ‘I think I have constipation’, 3000 persons were randomly extracted according to the population composition ratio of the Ministry of Internal Affairs and Communications Statistics Bureau metropolis and districts of Japan (Fig. 1).

The internet survey and statistical analysis of the data were both performed by Rakuten Insight Inc. (Osaka, Japan).

Diagnosis of IBS-C and functional constipation according to Rome III criteria.

The questionnaire about IBS-C and FC was prepared on the basis of Rome III criteria [10, 11]. According to Rome III, FC is diagnosed if two or more of the following symptoms have been present for at least 3 months (with symptom onset at least 6 months prior to diagnosis):

- Straining during at least 25% of defecations.
- Lumpy or hard stools in at least 25% of defecations.
- Sensation of incomplete evacuation for at least 25% of defecations.
- Sensation of anorectal obstruction/blockage for at least 25% of defecations.
- Manual maneuvers to facilitate at least 25% of defecations (e.g., digital evacuation, support of the pelvic floor).
- Fewer than three defecations per week.

In addition, loose stools are rarely present without laxatives and there are insufficient criteria to make a diagnosis of irritable bowel syndrome.

IBS is diagnosed if a person has had recurrent abdominal pain or discomfort at least 3 days per month in the last 3 months associated with two or more of the following:

1. Improvement with defecation.
2. Onset associated with a change in frequency of stool.
3. Onset associated with a change in form (appearance) of stool.

Criterion fulfilled for the last 3 months with symptom onset at least 6 months prior to diagnosis is also required.

For diagnosis of IBS-C, there must be constipation in addition to the above symptoms, with a variable number of the features of FC. The major difference between the two conditions is that abdominal pain occurs in IBS-C and FC is painless.

### Investigation of the demographic profile

The following demographic factors were investigated in all of the participants: age, gender, obstetric history (for women), height, body weight, BMI, annual income, educational history, occupation, stool frequency, and use of laxatives (including the place of purchase and the monthly cost).

### Investigation of symptoms, QOL, and mental symptoms

The severity of constipation was determined using the Bristol Stool Form Scale [12, 13], which classifies stools into 7 types based on appearance. Among these 7 morphologic types, Types 1 and 2 (Type 1; separate hard lumps, like nuts, hard to pass; Type 2: sausage-shaped, but lumpy) indicate constipation, Types 3 (like sausage, but with cracks on surface) and Types 4 (like sausage or snake, smooth and soft) and Types 5 (soft blobs with clear cut edges, passed early) are “ideal”, Types 6 (fluffy pieces with ragged edges, mushy stool) Types 7 (watery, no solid pieces) indicate diarrhea. The Japanese IBS severity index [14] (IBS-SI-J) was used for determination of the severity of IBS-C. In addition, the QOL of the participants was assessed by using the Japanese IBS QOL scale [15] (IBS-QOL-J), which has 38 items that are each answered on a 5-point scale (0: absent/no, 1: slightly, 2: moderately, 3: strongly, 4: very strongly). Health-related QOL, particularly physical and mental health, was investigated by using the SF-8 based on 8 subscales [physical functioning, role (physical), bodily pain, general health, vitality, social functioning, role (emotional), and mental health]. Mental symptoms were assessed by employing the Hospital Anxiety and Depression Scale (HADS) [16], which has 7 items related to depression and 7 items for anxiety.

### Investigation of lifestyle and diet

The influence of symptoms related to IBS-C and FC on the lifestyle of the participants was evaluated using the Japanese Health Practice Index (JHPI) [17]. Based on the Stanford University criteria [18], foods were classified into 17 food groups according to the content of fermentable oligosaccharides, disaccharides, monosaccharides and polyols (FODMAPs). Then the foods in the different food groups were divided into high-FODMAP and low-FODMAP foods, according to the previous report (Table 3) [19].

### Statistical analysis

The data are presented as mean ± standard deviation (SD) or median [interquartile range (IQR)]. In addition, the unpaired* t*-test or the 2-sample Wilcoxon rank sum test (Mann–Whitney *U* test) was used for inter-group comparison of numerical or ordinal scale data, as appropriate. Chi-square test or residual test was used for inter-group comparison of categorical data, as appropriate. In all analyses, the level of significance was set at 0.05 (two-sided). The Holm’s method was used to correct for the multiplicity of the test. The statistical analyses were performed using SPSS ver. 23.0 for Windows (IBM Japan, Ltd., Tokyo, Japan).

### Ethical considerations

This study was approved by the institutional review board of Aichi Medical University (October 6, 2016; approval no. 2016-H025). This study was carried out in conformity with the principles of the Declaration of Helsinki and the Ethical Guidelines for Medical and Health Research Involving Human Subjects enacted by the Japanese Ministry of Education, Culture, Sports, Science and Technology and the Ministry of Health, Labour and Welfare (December 22, 2014).

## Results

### Profile of the subjects

A total of 262 subjects (8.73% of the total survey population) were classified into the FC group, including 73 men (27.9%) and 189 women (72.1%). This group had a mean age of 49.8 ± 13.1 years and the mean BMI was 21.0 ± 3.3 kg/m^2^. Another 149 subjects (4.97% of the total survey population) were classified into the IBS-C group, including 76 men (51.0%) and 73 women (49.0%). The IBS-C group had a mean age of 41.6 ± 13.7 years and the mean BMI was 20.8 ± 3.0 kg/m^2^ (Table 1). While the FC group showed female predominance and was significantly older than the IBS-C group, there was no difference of BMI between the 2 groups. In addition, the IBS-C group included significantly more persons in their 20 s compared with the FC group (28.2% vs. 9.5%, *p* < 0.001), as well as significantly more persons in their 30 s (24.8% vs. 16.8%, *p* = 0.049). On the other hand, the FC group had significantly more persons in their 50 s than the IBS-C group (18.7 vs. 10.1%, *p* = 0.020) and also had significantly more persons in their 60 s (32.8% vs. 16.1%, *p* < 0.001). Overall, a significantly higher percentage of the IBS-C group was aged < 40 years compared with the FC group (53.0% vs. 26.3%, *p* < 0.001), while persons aged ≥ 40 years accounted for a larger percentage of the FC group (73.7% vs. 47.0%) and the majority of the participants in this group were elderly (Table 1). There were no significant differences of the places of residence between the two groups, and there was a tendency for persons from the IBS-C group to be more likely to live in urban areas such as the Kanto area (IBS-C group: 38.3% vs. FC group: 35.5%), the Kinki area (IBS-C group: 24.2% vs. FC group: 22.9%), and the Chubu area (IBS-C group: 21.5% vs. FC group: 13.4%), while persons from the FC were more likely to live in rural areas such as Hokkaido (FC group: 4.6% vs. IBS-C group: 2.7%), Tohoku (FC group: 8.0% vs. IBS-C group: 2.7%), and Kyushyu (FC group: 9.2% vs. IBS-C group: 3.4%). There were no significant differences of the educational background between the two groups. However, the IBS-C group had a slightly higher level of academic qualifications than the FC group, with 34.7% of the FC group finishing their education at the high school level compared with 36.9% of the IBS-C group. There were also no significant differences of the annual income, which was < 6 million yen and ≥ 6 million yen in a similar proportion of both the groups. Finally, there were no significant differences of occupation. In both the groups, the most frequent occupation was office worker/public servant and this was followed by part time worker in the IBS-C group and by home duties in the FC group (Table 1).

**Table 1:** Profile of the subjects

	FC group (*n* = 262)	IBS-C group (*n* = 149)	*P* value
Age (years)	49.8 ± 13.1	41.6 ± 13.7	< 0.001* (c)
Age group (*n*, %)			
20 s	25, 9.5%	42, 28.2%	< 0.001* (b)
30 s	44, 16.8%	37, 24.8%	0.049* (b)
40 s	58, 22.1%	31, 20.8%	0.753 (b)
50 s	49, 18.7%	15, 10.1%	0.020* (b)
60 s	86, 32.8%	24, 16.1%	< 0.001* (b)
< 40	69, 26.3%	79, 53.0%	< 0.001* (a)
≥ 40	193, 73.7%	70, 47.0%	
Delivery (*n*, %)	115, 60.8%	28, 38.4	0.001* (a)
Height (cm)	161.5 ± 8.6	164.2 ± 8.6	0.002* (c)
Body weight (kg)	55.2 ± 12.1	56.4 ± 11.2	0.309 (c)
BMI (kg/m^2^)	21.0 ± 3.3	20.8 ± 3.0	0.507 (c)
Residence (*n*, %)			0.042* (a)
Kanto	93, 35.5%	57, 38.3%	
Kinki	60, 22.9%	36, 24.2%	
Kyusyu	24, 9.2%	5, 3.4%	
Shikoku	7, 2.7%	6, 4.0%	
Chugoku	10, 3.8%	5, 3.4%	
Chubu	35, 13.4%	32, 21.5%	
Tohoku	21, 8.0%	4, 2.7%	
Hokkaido	12, 4.6%	4, 2.7%	
Highest educational level (*n*, %)			0.060 (a)
Junior high school	3, 1.1%	6, 4.0%	
High school	91, 34.7%	55, 36.9%	
Technical college	19, 7.3%	10, 6.7%	
Vocational school	16, 6.1%	15, 10.1%	
Junior college	43, 16.4%	10, 6.7%	
University	81, 30.9%	47, 31.5%	
Graduate school	8, 3.1%	6, 4.0%	
Others	1, 0.4%	0, 0.0%	
Classification by educational level (*n*, %)			0.323 (a)
Junior high-vocational school	94, 36.0%	61, 40.9%	
Junior college-graduate school	167, 67.0%	88, 59.1%	
Annual income (*n*, %)			0.886 (a)
< 6 million yen	128, 59.8%	72, 59.0%	
≥ 6 million yen	86, 40.2%	50, 41.0%	
[No answer (*n*)%]	[48]	[27]	
Occupation (*n*, %)			< 0.001* (a)
Student	1, 0.4%	6, 4.0%	
Office worker/public servant	103, 39.3%	59, 39.6%	
Self-employed/freelance	21, 8.0%	19, 12.8%	
Part time	36, 13.7%	29, 19.5%	
Retired/unemployed	27, 10.3%	15, 10.1%	
Home duties	72, 27.5%	18, 12.1%	
Others	2, 0.8%	3, 2.0%	

Data are expressed n and %, or mean ± standard deviation

*P* value: a: Chi-square test, b: residual analysis, c: unpaired *t*-test. *P-value < 0.05

### Defecation

The frequency of passing stools was less than 3 times per week in a significantly higher percentage of persons from the FC group than the IBS-C group (51.5% vs. 44.3%, *p* < 0.05) (see Supplementary Fig. 1). While a similar proportion of respondents in both the groups passed stools less than once a week or twice a week, only 8.7% of the IBS-C group passed stools once a week versus 13.7% of the FC group. There were no significant differences of the Bristol scale between the two groups, with hard stools of Types 1–2 being frequent in both the groups (about 40% of bowel motions) and normal to diarrhea stools accounting for 25–28% of bowel motions in both the groups (see Supplementary Fig. 1).

### Laxatives

**Fig. 2:**
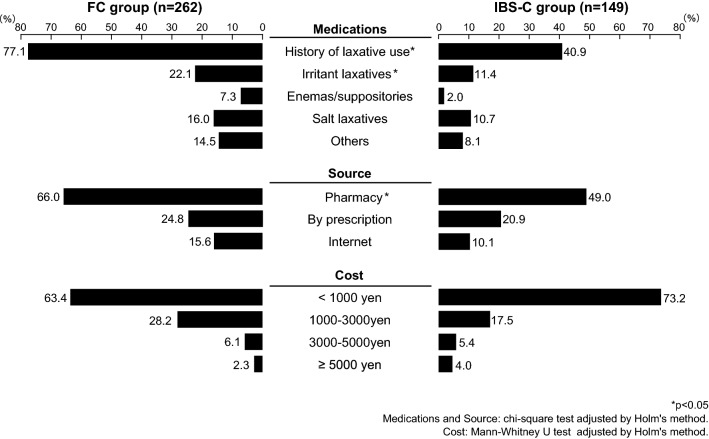
Comparison of medication of constipation, how to get drugs, and monthly cost. *p < 0.05. Medications and source: chi-square test adjusted by Holm's method. Cost: Mann–Whitney *U* test adjusted by Holm's method

When use of laxatives was investigated, significantly fewer persons used laxatives in the IBS-C group compared with the FC group (40.9% vs. 77.1%, *p* < 0.05). In addition, significantly fewer persons used irritant laxatives in the IBS-C group (11.4% vs. 22.1%, *p* < 0.05), but there was no significant difference between the two groups with regard to the use of salt laxatives (Fig. 2).

With respect to the source of laxatives, these were significantly more frequently purchased at a pharmacy by respondents from the FC group than by respondents from the IBS-C group (66.0% vs. 49.0%, *p* < 0.05). When the monthly cost of laxatives was investigated, a cost of less than 1000 yen was the most common answer given in both the groups. It was found that persons who spent 5000 yen or more on laxatives were slightly more frequent in the IBS-C group, but the difference was not significant (Fig. 2).

### Physical symptoms, QOL, and mental symptoms

**Fig. 3:**
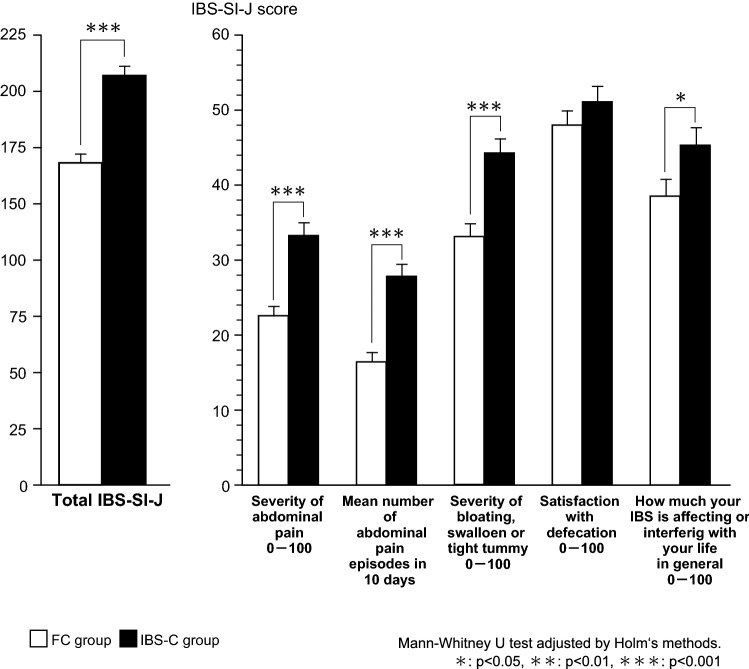
Comparison of IBS-SI-J between the FC and the IBS-C groups. Mann–Whitney *U* test adjusted by Holm's methods. *p < 0.05, **p < 0.01, ***p < 0.001

When the IBS-SI-J was assessed, the total score was significantly higher in the FC group than in the IBS-C group (*p* < 0.001) (Fig. 3). In addition, the scores for symptoms having an influence on daily life were all significantly higher in the IBS-C group compared with the FC group, including the severity of abdominal pain (*p* < 0.001), the frequency of abdominal pain (*p* < 0.001), the severity of bloating, swollen or tight tummy (*p* < 0.001), and the how much IBS affecting or interfering with your life in general (*p* < 0.05). With respect to the IBS-SI-J, both the total score and the frequency of moderate or severe symptoms were significantly higher in the IBS-C group than the FC group (202.4 ± 89.2 vs. 159.3 ± 80.0 and 56.7% vs. 44.6%, both *p* < 0.05).

Concerning the severity of evacuation difficulties as specified by the Rome III criteria, the frequency of 3 items (“Straining during at least 25% of defecations”, “Lumpy or hard stools in at least 25% of defecations”, and “Sensation of anorectal obstruction/blockage for at least 25% of defecations”) was significantly higher in the IBS-C group than the FC group (100% vs. 80.5%, 100% vs. 80.2% and 100% vs. 97.3%, respectively, all *p* < 0.001). On the other hand, the frequency of “Manual maneuvers to facilitate at least 25% of defecations (e.g., digital evacuation, support of the pelvic floor)” was significantly higher in the FC group than the IBS-C group (6.9% vs. 0.0%, *p* < 0.001). There was no significant difference in the frequency of a “Sensation of incomplete evacuation for at least 25% of defecations” between the groups, but it tended to be noted more often in the IBS-C group than the FC group (90.6% vs. 87.8%).

**Fig. 4:**
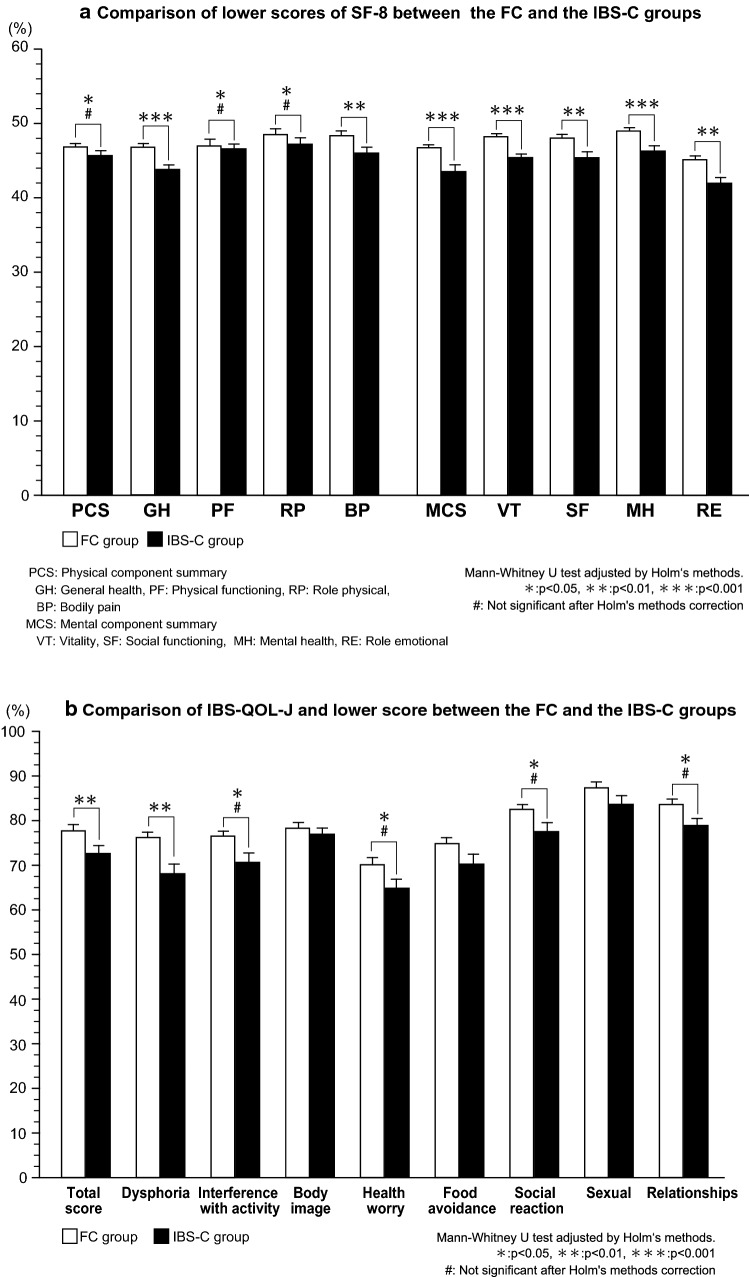
**a** Comparison of lower scores of SF-8 between the FC and the IBS-C groups. *PCS* physical component summary, *GH* general health, *PF* physical functioning, *RP* role physical, *BP* bodily pain, *MCS* mental component summary, *VT* vitality, *SF* social functioning, *MH* mental health, *RE* role emotional. Mann–Whitney *U* test adjusted by Holm's methods. *p < 0.05, **p < 0.01, ***p < 0.001. ^#^Not significant after Holm's methods correction. **b** Comparison of IBS-QOL-J and lower score between the FC and the IBS-C groups. Mann–Whitney *U* test adjusted by Holm's methods. *p < 0.05, **p < 0.01, ***p < 0.001. ^#^Not significant after Holm's methods correction

When the SF-8 was investigated, it was found that the scores for physical component summary (PCS) (*p *< 0.05) and mental component summary (MCS) (*p *< 0.0001) were significantly lower in the IBS-C group than in the FC group. However after adjustment by Holm’s method, PCS was not significant. In addition, except physical functioning (PF) and role physical (RP), the scores for other subscales were significantly lower in the IBS-C group than in the FC group. (Fig. 4a). With respect to the IBS-QOL-J, scores for the following components were significantly higher in the FC group: dysphoria (*p *< 0.01), interference with activity, (*p *< 0.05), health worry (*p *< 0.05), social reaction (*p *< 0.05), relationships (*p *< 0.05). However, there were no significant differences between the two groups regarding the scores for body image, food avoidance, and sexual problems. However, after adjustment by Holm’s method, only total score (*p *< 0.05) and dysphoria (*p *< 0.01) were significantly lower in the IBS-C group than in the FC group (Fig. 4b). On the other hand, the total HADS anxiety score and the total depression score were both significantly higher in the IBS-C group compared with the scores in the FC group (both *p *< 0.001). However, although positive rate of anxiety was significantly higher in the IBS-C group, no significant difference was noted with depression between both the groups (see Supplementary figure 2 and Supplementary table 1).

### Lifestyle and FODMAP intake

The JHPI lifestyle survey [17] revealed that the frequency of getting ‘enough sleep’ was significantly lower in the IBS-C group than the FC group (*p* = 0.016) (Table 2). Although persons taking sedatives/hypnotics were excluded from this survey to avoid drug-induced constipation, it is interesting that fewer than half of the respondents in either group were able to obtain sufficient sleep. On the other hand, lifestyle factors such as smoking and drinking alcohol did not show a significant difference between the two groups, and neither did items related to exercise. There were also no significant differences of items related to weight gain and items related to eating habits between the IBS-C group and the FC group.

**Table 2:** Comparison of habits as shown by the JHPI interview

JHPI interview	FC group (*n* = 262)	IBS-C group (*n* = 149)	*P* value
Smoking (100/month or for ≥ 6 months)	41, 15.6%	18, 12.1%	0.321 (a)
Drinking alcohol			0.880 (b)
No	106, 40.5%	55, 36.9%	
Sometimes	103, 39.3%	73, 49.0%	
Daily	53, 20.2%	21, 14.1%	
Daily alcohol consumption			0.320 (a)
180 mL sake	93, 59.6%	50, 53.2%	
1 bottle of beer	63, 40.4%	44, 46.8%	
Weight gain ≥ 10 kg compared with 20 years old	45, 17.2%	27, 18.1%	0.809 (a)
Exercise for > 30 min ≥ twice a week for more than 1 year	57, 21.8%	28, 18.8%	0.476 (a)
Daily walking or equivalent physical activity for 1 h	71, 27.1%	44, 29.5%	0.598 (a)
Walks faster than other people of the same age	90, 34.4%	55, 36.9%	0.601 (a)
Weight gain ≥ 3 kg during the past 1 year	86, 32.8%	57, 38.3%	0.266 (a)
Eats faster than other people	165, 63.0%	88, 59.1%	0.433 (a)
Dinner within 2 h before sleep ≥ 3 times a week	67, 25.6%	42, 28.2%	0.564 (a)
Snack after dinner ≥ 3 times a week	83, 31.7%	52, 34.9%	0.504 (a)
Skips breakfast ≥ 3 times a week	69, 26.3%	49, 32.9%	0.158 (a)
Getting enough sleep	118, 45.0%	49, 32.9%	0.016 (a)

*P* value: a: Chi-square test, b: Mann–Whitney *U* test

Data are expressed *n* and %

Regarding diet, the frequency of eating certain high-FODMAP foods was significantly higher in the FC group than the IBS-C group, including bread (wheat or rye) and fruits (apples, pears, apricots, and watermelon) (Table 3). The frequency of eating certain low-FODMAP foods was also significantly higher in the FC group than the IBS-C group, including some fruits (mandarins, bananas, and strawberries), some vegetables (spinach, carrots and potatoes) and hard cheese. On the other hand, intake of low-FODMAP grains such hard cheese was significantly higher in the IBS-C group than the FC group. The intake of isomerized sugar was also significantly higher in the IBS-C group compared with the FC group.

**Table 3:** Intake of high- and low-FODMAP foods

	FC group	IBS-C group	*P* value
High-FODMAP foods			
Milk/yogurt	4 (3–5)	4 (2–5)	0.253
Ripe cheese (gorgonzola, blue cheese)	2 (1–3)	2 (1–2)	0.141
Apples, pears, apricots, watermelon, dried fruit	3 (2–4)	2 (2–4)	< 0.001*
Bread (wheat, rye)	4 (3–5)	4 (3–4)	0.050*
Pasta	3 (2–4)	3 (2–4)	0.700
Beans	4 (3–4)	3 (2–4)	0.253
Pistachios, cashew nuts	2 (2–3)	2 (2–3)	0.364
Ketchup, dressing	3 (2–4)	4 (3–4)	0.630
Soda drinks, high fructose syrup, honey	2 (2–3)	2 (2–3)	0.095
Instant coffee	4 (2–5)	4 (2–5)	0.631
Xylitol gum	2 (1–3)	2 (1–3)	0.961
Boil-in-the-bag prepared foods (isomerized sugar)	3 (2–3)	3 (2–4)	0.011*
Low-FODMAP foods			
Hard cheese	2 (1–3)	2 (1–3)	0.016*
Mandarins, bananas, strawberries	3 (2–4)	3 (2–4)	0.022*
Spinach, carrots, potatoes	4 (3–4)	3 (2–4)	0.040*
Rice, cereals	4 (4–5)	4 (4–5)	0.516
Refined sugar	4 (2–4)	4 (3–4)	0.769

Data are expressed median (inter quartile range)

*P* value: Mann–Whitney *U* test, *P < 0.05

## Discussion

In 2017, clinical practice guidelines for constipation were published in Japan [19], so it is hoped that this condition will attract increased recognition and that new evidenced-based treatments will be developed. The present survey was performed to investigate the prevalence of IBS-C and FC as defined by Rome III criteria [2] among persons with constipation who fitted the demographic profile of the general Japanese population under 70 years old.

Heidelbaugh et al. previously reported that the prevalence of IBS-C and FC by Rome III criteria [2] in the USA was 3.3% and 5.5%, respectively [5]. Generally, the prevalence of IBS-C has been reported to be approximately 12% in Europe and the USA [20], while the reported prevalence ranges from 7 to 17% in Asia [20]. According to Saito et al. [21], IBS has a prevalence of 14.2% in the general Japanese population, with a 1-year morbidity rate of 1–2%, while another study showed that its prevalence was a high 31% among the outpatients of internal medicine departments [22]. Evaluation of FC by the Rome criteria (I, II, and III) has not been well documented because this concept was not clear in Rome I. With respect to IBS-C, a meta-analysis showed that the prevalence of IBS according to the Rome I, Rome II, and Rome III criteria was 8.8%, 9.4%, and 12.2%, respectively [20]. Thus, sensitivity increased across the criteria, suggesting that caution should be exercised when evaluating the prevalence of IBS-C.

Between one-quarter and one-third of IBS patients are thought to have IBS-C, corresponding to around 4.7% of the general population, which seems to be similar to our result regarding the prevalence of IBS-C. In the present study, we excluded persons with secondary constipation or drug-induced constipation, which may explain the lower prevalence of constipation than in other reports. In general, it has been reported that the prevalence of constipation increases with aging [1, 23], while the prevalence of IBS decreases [24]. In the present study, we found that a significantly higher percentage of the IBS-C group was aged < 40 years compared with the FC group, while a significantly higher percentage of the FC group was formed by persons who were ≥ 40 years old. A systematic review performed by Lovell et al. showed a decrease in the prevalence of IBS along with aging [20]. Accordingly, it can be suggested that the pathogenesis of IBS may be age-related or strongly influenced by age, but further studies will be needed to elucidate this potential relationship.

It has been reported that the prevalence of constipation is lower among persons with a higher socioeconomic status [3, 21], while the opposite trend has been identified for IBS [25]. However, we could not find any significant differences of socioeconomic status between the IBS-C group and the FC group in the present study, possibly because there is less disparity of annual income and educational background in Japan than in Europe or the USA. There were also no significant differences of the place of residence between the two groups, although respondents from the IBS-C group were more likely to live in urban areas such as the Kanto, Chubu, and Kinki areas than respondents from the FC group, while those from the FC group were more likely to live in rural areas such as Hokkaido, Tohoku, and Kyushyu. Because IBS is more frequent among people living in cities, there is a possibility that the prevalence of IBS-C would also be higher in larger cities where life is more stressful. To confirm this, it would be necessary to not only investigate the geographical place of residence, but also the population of the cities or towns in which the respondents lived.

With respect to items regarding lifestyle from the JHPI [17], the percentage of respondents who answered ‘I eat faster than other people’ was higher in the FC group and the percentage who answered ‘I tend to skip breakfast’ was higher in the IBS-C group. According to a previous investigation of the characteristics associated with constipation, skipping breakfast is significantly more frequent among persons with constipation than among healthy persons [26] In agreement with this report, we found that persons in the IBS-C group skipped breakfast more often than those in the FC group. Diarrhea associated with IBS is often more frequent in the morning than the nighttime, which can affect commuting to work or going to school, and it has been reported that IBS patients tend to skip breakfast in order to avoid diarrhea [27]. Our study identified the same behavior in IBS-C patients who show predominance of constipation over diarrhea, but it is possible that they skipped breakfast to avoid aggravation of abdominal discomfort.

We also found that use of laxatives was low in the IBS-C group. A stool frequency < 3 times a week, which is important in the Rome III criteria, was significantly less common in the IBS-C group than in the FC group, so it is possible that the respondents with IBS-C may have considered laxatives were not necessary. Alternatively, they may have thought that use of laxatives could aggravate their symptoms.

It has been reported that the role of psychological factors in IBS increases along with the severity of this condition [1]. Typical psychological abnormalities associated with IBS are reported to be depression and anxiety, followed by somatization [28]. In addition, stress during early life was reported to be a risk factor for the development of IBS [29], and it has been found that patients with IBS display catastrophe-oriented thinking and show digestive tract-specific anxiety [29]. Many authors have described the existence of a relation between psychological abnormalities and constipation. IBS-C patients have significantly more upper abdominal symptoms than diarrhea-related symptoms, and several previous studies have demonstrated that QOL is significantly worse when IBS is accompanied by upper abdominal symptoms [24, 30, 31]. According to a study performed in the USA, both anxiety disorder (odds ratio: 3.02) and depression (odds ratio: 2.31) were significantly more frequent among IBS patients than among age- and gender-matched controls [32]. Likewise, a systematic review of 10 case–control studies performed in Europe comparing healthy subjects with IBS patients demonstrated a significantly higher prevalence of anxiety and depression among the IBS patients [25]. According to our findings in the present study, QOL was reduced by constipation and the existence of constipation was closely related to stress. We also showed that some parameters of QOL was significantly worse in the IBS-C group compared with the FC group. Therefore, it is considered that careful evaluation of stress and maintaining good relations with patients should form the basis of medical care for persons who have constipation or IBS [33].

With respect to diet, interesting results were obtained through comparison of the intake of high- and low-FODMAP foods by the two groups [34]. In the IBS-C group, intake of certain high-FODMAP foods, including fruits (such as apples, pears, apricots, and watermelon) and bread was lower than in the FC group, as was the intake of certain low-FODMAP foods (hard cheese, mandarins, bananas and some vegetables including spinach, carrots and potatoes). It has been reported that low-FODMAP foods are associated with less abdominal distention and are effective for diarrhea in persons with IBS, but the effect of such foods on constipation is unknown [24, 28–30, 35].The results obtained in the present study suggested that persons with IBS-C may empirically select foods that are less likely to cause abdominal symptoms such as bloating and abdominal pain. On the other hand, we found that persons with FC preferred high-FODMAP foods that could improve their bowel movements and were not so concerned about the potential risk of developing abdominal symptoms.

## Limitations

In 2016, validation of the Japanese version of the Rome IV criteria was not completed, so we could not use Rome IV criteria. So, we had to use the Japanese version of the Rome III criteria, which had been validated. If we could use the Rome IV criteria, the results of our survey may become somewhat different.

Because this study was based on an internet survey, there is some risk that the data obtained are unreliable. However, we concluded that this survey was likely to be sufficiently reliable because the respondents were from a registered panel and the identity of each participant was confirmed by a research company. This survey involved 3000 subjects who were randomly extracted to match the profile of the general Japanese population [1]. Because the mean age of the panel was less than 70 years, different results may have been obtained if an older panel had been interviewed. However, the age range of the present panel was considered to be appropriate for research on functional gastrointestinal disease, especially IBS [31]. While internet surveys have certain limitations, there is the advantage that data on many parameters can be obtained directly from the subjects themselves in a short time without requiring any intervention by healthcare workers. Thus, internet surveys seem to be useful for performing cross-sectional studies from the perspective of obtaining patient-reported outcomes without the risk of bias due to data collection by health care personnel.

## Conclusions

Among Japanese persons with constipation aged under 70 years-old, an internet survey demonstrated that the prevalence of FC and IBS-C conforming to Rome III criteria was 8.73% and 4.97%, respectively. Compared with the respondents who had FC, those with IBS-C were found to be younger and to have more severe symptoms, along with worse QOL and a higher prevalence of anxiety and depression. Respondents with FC or IBS-C also showed differences in relation to their lifestyle and diet, including the intake of high- and low-FODMAP foods, as well as differences regarding the management of constipation, with use of laxatives being significantly more frequent in the FC group. It is hoped that these findings will prove useful for increasing our understanding of this common and troublesome condition, as well as providing some clues to improve its management.

## Electronic supplementary material

Below is the link to the electronic supplementary material.

Supplementary file1 (PDF 293 kb)

Supplementary file2 (DOCX 15 kb)
