# Supplementary material for: Internet survey on the actual situation of constipation in the Japanese population under 70 years old: focus on functional constipation and constipation-predominant irritable bowel syndrome
Source: J Gastroenterol. 2019 Aug 19;55(1):27–38. doi: 10.1007/s00535-019-01611-8 (PMC6942565; doi:10.1007/s00535-019-01611-8)

Supplementary figure 1
Comparison of stool frequency and shape between IBS-C group and FC Group

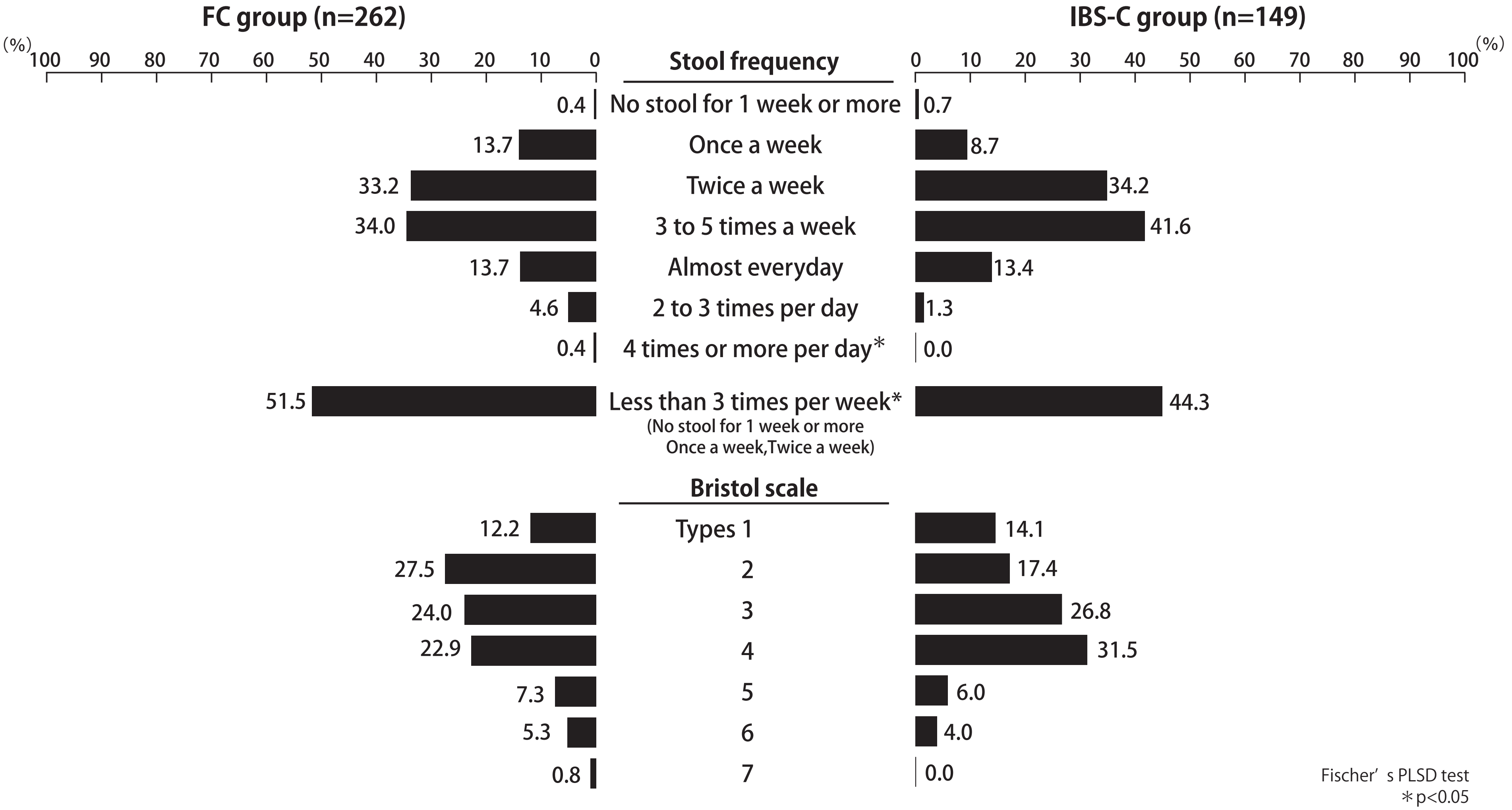

Supplementary figure 2
Assessment by HADS

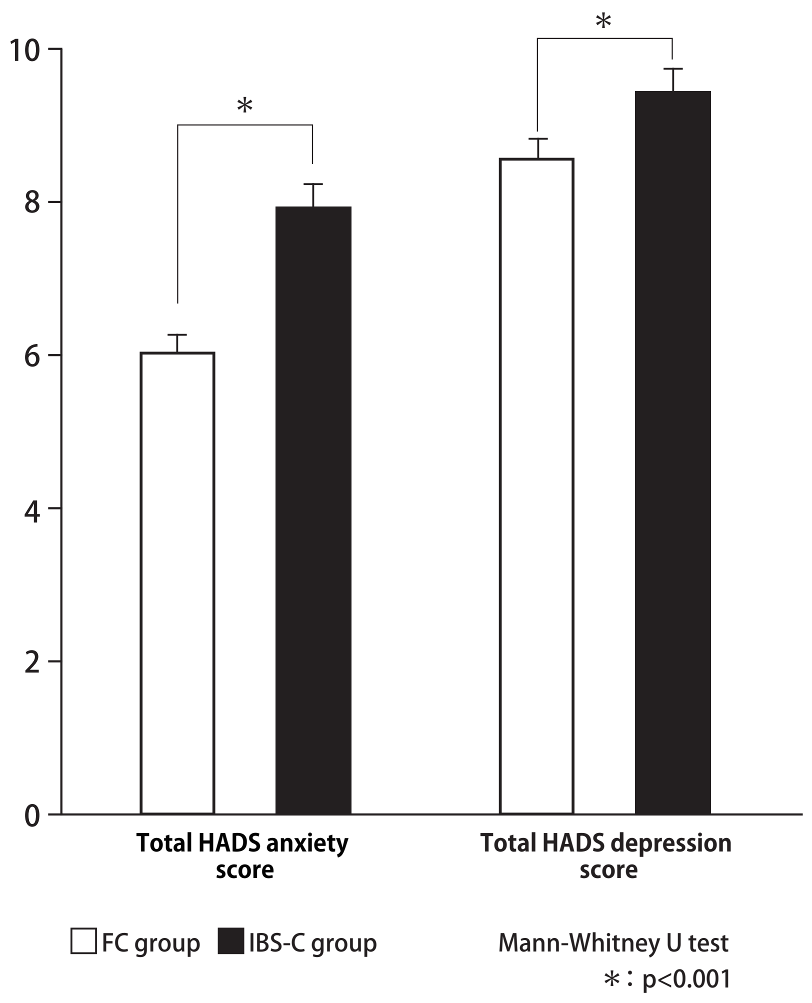

Supplement: Supplementary file 1 — Supplementary file1 (PDF 293 kb) [file 535_2019_1611_MOESM1_ESM.pdf]
