# Supplementary material for: Internet survey on the actual situation of constipation in the Japanese population under 70 years old: focus on functional constipation and constipation-predominant irritable bowel syndrome
Source: J Gastroenterol. 2019 Aug 19;55(1):27–38. doi: 10.1007/s00535-019-01611-8 (PMC6942565; doi:10.1007/s00535-019-01611-8)
Supplement: Supplementary file 2 — Supplementary file2 (DOCX 15 kb) [file 535_2019_1611_MOESM2_ESM.docx]

|  |
| --- |

Supplementary Table 1.

Comparison of anxiety and depression positive rates between the FC and IBS-C groups

|  | FC group (n=262) | IBS-C group  (n=149) | *p* value |
| --- | --- | --- | --- |
| Anxiety scale  < 7  ≧7 | 153 (58.4%)  109 (41.6%) | 59 (39.6%)  90 (60.4%) | <0.0001 |
| Depression scale  < 7  ≧7 | 79 (30.2%)  183 (69.8%) | 38 (25.5%)  111 (74.5%) | 0.363 |

*p* value：Fisher's exact test.　Result is determined positive 7 or more.
